# 

**Published:** 1909-01-09

**Authors:** 


### GRADUATES' MEDICAL SCHOOLS.

DIARY FOR WEEK JAN. 11 TO JAN. 16.

### HOSPITAL FOR CONSUMPTION AND DISEASES OF THE CHEST, Brompton, S.W.

**At 4 p.m.**
**Jan. 13,** Mr. Stanley Boyd, **Difficult Empyemata.**

### ST. JOHN'S HOSPITAL FOR DISEASES OF THE SKIN, Leicester Square, W.C.

**At 6 p.m.**
**Jan. 14,** Chesterfield Lectures, **Herpes Zoster, Dermatitis Herpetiformis.**

### THE POST-GRADUATE COLLEGE, West London Hospital, Hammersmith, W.

**At 10 a.m.**
**Jan. 11 and 14,** Surgical Registrar, **Demonstration of Cases in the Wards.**
**Jan. 15,** Medical Registrar, **Demonstration of Cases in the Wards.**
**At 12 noon.**
**Jan. 11,** Dr. Low, **Pathological Demonstration.**
**At 12.15 p.m.**
**Jan. 13 and 16,** Dr. Pritchard, **Practical Medicine.**
**At 5 p.m.**
**Jan. 11,** Dr. Saunders, **Clinical.**
**Jan. 12,** Dr. Low, **Blood Examination in Tropical Diseases.**
**Jan. 13,** Mr. Baldwin, **Practical Surgery.**
**Jan. 14,** Mr. Bidwell, **Displaced Kidney.**
**Jan. 15,** Mr. Armour, **Clinical.**

### BOOKS RECEIVED.

J. AND A. CHURCHILL.

"Reports of the Society for the Study of Disease in Children." Vol. VIII.

THE CELTIC PRESS.

"Infected Ears" (Intrameatal Treatment). By F. Faulder White, F.R.C.S.

E. J. ARNOLD AND SON, LTD., LEEDS.

"Some Characteristics and Requirements of Childhood." By Alice Ravenhill, F.R.San.I.

J. BALE, SONS, AND DANIELSSON, LTD.

"Hints to Ships' Surgeons." By J. F. Elliott, L.R.C.S.

KEGAN PAUL, TRENCH, TRUBNER, AND CO., LTD.

"Human Speech." By N. C. Macnamara, F.R.C.S.

OLIVER AND BOYD, EDINBURGH.

"Practical Gynæcology." By Netta Stewart and J. Young, M.B.

REYNOLDS-BALL'S GUIDES.

"The Levantine Riviera." By W. T. Beeby, M.D., and E. Reynolds-Ball, F.R.G.S.

THE SCIENTIFIC PRESS, LTD.

"Syllabus of Lectures on Home Nursing." By A. Birch.

A. AND C. BLACK.

"The Englishwoman's Year Book and Directory, 1909."
"Who's Who, 1909."
"The Writers' and Artists' Year Book, 1909."

J. WRIGHT AND CO., BRISTOL.

"Index of Treatment." Fourth edition. Edited by Robert Hutchison, M.D., F.R.C.P., and H. Stansfield Collier, F.R.C.S.

### ANSWERS TO CORRESPONDENTS.

H. BARRY.—The following official statement, referring to medical practice in the United Kingdom, answers your question: " All persons whose registration is in respect of any Diploma or Diplomas dated after June 30, 1887, are persons legally qualified for the practice of Medicine, Surgery, and Midwifery."

CHARLES DICKENS presided at the festival dinner in aid of the Royal Hospital for Incurables, Putney Heath, in the year 1854, and on January 6 of this year his grandchildren and their friends gave an entertainment to the patients of the same hospital, as they have done in former years.

THE Gas Light and Coke Company announce a further reduction in the price of gas. This time last year they reduced their price from 2s. 11d. to 2s. 10d. per 1,000 cubic feet, and now a further drop to 2s. 9d. is announced. Gas is now so widely used in the home, not merely for lighting, but also as a cleanly and convenient substitute for coal, that this steady decrease in price is of considerable importance to a great many people, and will undoubtedly lead to a still greater consumption of gas for fuel, both in the home and in the workshop and factory

THE HOSPITAL JANUARY 9, 1909.

*Name* ..............................................

*Address* ..............................................

..............................................

**This Coupon must accompany manuscript or contributions intended for THE HOSPITAL.**

